# Chloro­bis­(naphthalen-1-yl)phosphane

**DOI:** 10.1107/S1600536811033782

**Published:** 2011-08-31

**Authors:** Ana Foi, Sebastian A. Suarez, Fabio Doctorovich

**Affiliations:** a Departamento de Química Inorgánica, Analítica y Química Física/INQUIMAE-CONICET, Facultad de Ciencias Exactas y Naturales, Universidad de Buenos Aires, Argentina

## Abstract

In the title compound, C_20_H_14_ClP, the dihedral angle between the naphthyl rings is 81.77 (6)°. The crystal packing suggests weak π–π stacking inter­actions between the naphthyl rings in adjacent units [minimum ring centroid separation 3.7625 (13) Å].

## Related literature

For the structure of a similar compound, see: Schiemenz *et al.* (2003 ▶). For details of the synthetic procedures, see: Wesemann *et al.* (1992 ▶).
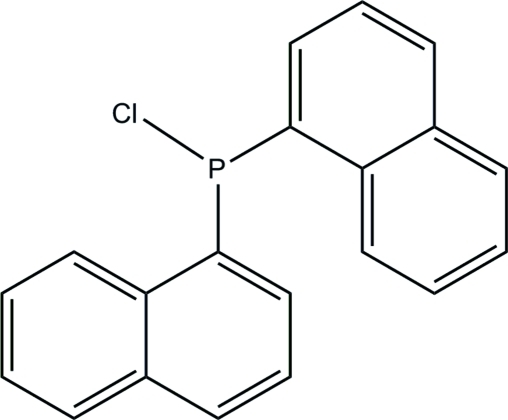


## Experimental

### 

#### Crystal data

C_20_H_14_ClP
*M* *_r_* = 320.76
Monoclinic, 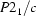

*a* = 12.4335 (6) Å
*b* = 10.4510 (4) Å
*c* = 11.9293 (7) Å
β = 93.180 (5)°
*V* = 1547.74 (13) Å^3^
*Z* = 4
Mo *K*α radiation
μ = 0.34 mm^−1^
*T* = 298 K
0.30 × 0.20 × 0.10 mm

#### Data collection

Oxford Gemini E CCD diffractometer
8306 measured reflections
3531 independent reflections
1723 reflections with *I* > 2σ(*I*)
*R*_int_ = 0.041

#### Refinement

*R*[*F* ^2^ > 2σ(*F* ^2^)] = 0.039
*wR*(*F* ^2^) = 0.079
*S* = 0.78
3531 reflections
199 parameters
H-atom parameters constrained
Δρ_max_ = 0.24 e Å^−3^
Δρ_min_ = −0.22 e Å^−3^

### 

Data collection: *CrysAlis PRO* (Oxford Diffraction, 2009 ▶); cell refinement: *CrysAlis PRO*; data reduction: *CrysAlis PRO*; program(s) used to solve structure: *SHELXS86* (Sheldrick, 2008 ▶); program(s) used to refine structure: *SHELXL97* (Sheldrick, 2008 ▶); molecular graphics: *ORTEP-3 for Windows* (Farrugia, 1997 ▶); software used to prepare material for publication: *WinGX* (Farrugia, 1999 ▶).

## Supplementary Material

Crystal structure: contains datablock(s) global, I. DOI: 10.1107/S1600536811033782/zs2136sup1.cif

Structure factors: contains datablock(s) I. DOI: 10.1107/S1600536811033782/zs2136Isup2.hkl

Additional supplementary materials:  crystallographic information; 3D view; checkCIF report

## Figures and Tables

**Fig. 1.**
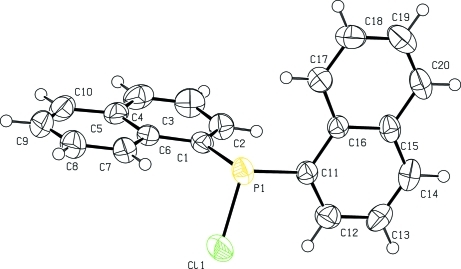
Molecular conformation and atom numbering scheme for the title compound. Displacement ellipsoids are drawn at the 50% probability level.

**Fig. 2.**
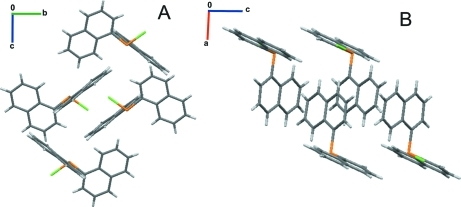
Crystal packing for the title compound viewed along a (A) and along b (B)

**Fig. 3.**
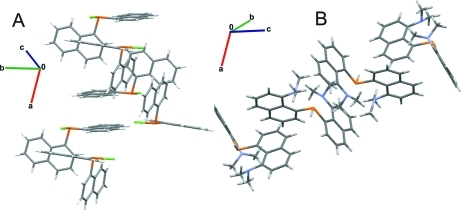
Packing arrangement for different bis(1-naphthyl)phosphines. A = the title compound; B = bis(8-diethylaminonaphth-1-yl)phosphine.

## supplementary crystallographic information

### Comment

The title compound, C_20_H_14_ClP, was obtained in the course of our continuing studies on the synthesis of phosphonium salts, with the aim of using them as building blocks in crystal engineering. In the structure (Fig. 1), the dihedral angle between the naphthyl rings is 81.77 (6)°, corresponding to torsion angles C1—P1—C11—C12 and C11—P1—C1—C2 of 100.58 (18)° and 2.03 (18)° respectively while an intramolecular C—H···Cl hydrogen-bonding interaction [C12—H12···Cl1, 3.128 (2) Å] stabilizes the conformation of one of the naphthyl rings [torsion angle Cl1—P1—C11—C12 = -1.68 (18)°]. Both of these naphthyl ring systems are essentially planar, with mean deviations from their least-square planes of 0.071 (2) Å for the C1–C10 system and 0.021 (2) Å for the C11–C20 system. The structural analysis of the title compound shows no significant bond differences compared to those found in similar structures, e.g. the P—Cl distance [2.0867 (8) Å *cf.* 2.10 (6) Å] and the P—C distances [P1—C1, 1.8294 (18) Å and P1–C11, 1.8309 (19) Å] comparing with 1.84 (3) Å.

A comparison with the previously reported structure of bis(8-diethylaminonaphth-1-yl)phosphine (Schiemenz *et al.*, 2003) which shows no evidence of π–π stacking interactions, differs from that of the title compound which shows weak interactions between the naphthalene rings in adjacent molecules [minimum ring centroid separation, 3.7625 (13) Å]. It is likely that due to the presence of Cl instead of the group N(CH_3_)_2_ there is less steric repulsion between the substituents, which is evidenced by a smaller separation between the naphthyl moieties, allowing the π–π interactions between the aromatic rings to take place.

### Experimental

The title compound was obtained as a by product in the synthesis of tris(1-naphthyl)phosphine (Wesemann *et al.*, 1992). The synthesis was carried out in two steps. 7.27 mmol of 2-bromonaphthalene and 7.37 mmol of *n*-butyllithium were added to 20 ml of diethyl ether at -30°C, in order to obtain the naphthyllithium intermediate. 2.4 mmol of PCl_3_ dissolved in 10 ml of diethyl ether were added to the reaction mixture and refluxed for 2 h. The by product chlorobis(1-naphthyl)phosphine was separated from the major product of the synthesis (tris(1-naphthyl)phosphine), after recrystallization of the reaction mixture from toluene.

### Refinement

Several H atoms were detected at approximate locations in a difference Fourier map. Subsequently, however, they were positioned stereochemically and refined using a riding model, with C—H = 0.93 Å and *U*_iso_(H) = 1.2*U*_eq_(C).

### Crystal data

**Table:**

C_20_H_14_ClP	*F*(000) = 664
*M**_r_* = 320.76	*D*_x_ = 1.377 Mg m^−^^3^
Monoclinic, *P*2_1_/*c*	Mo *K*α radiation, λ = 0.71073 Å
Hall symbol: -P 2ybc	Cell parameters from 2101 reflections
*a* = 12.4335 (6) Å	θ = 3.6–28.6°
*b* = 10.4510 (4) Å	µ = 0.34 mm^−^^1^
*c* = 11.9293 (7) Å	*T* = 298 K
β = 93.180 (5)°	Prism, colourless
*V* = 1547.74 (13) Å^3^	0.30 × 0.20 × 0.10 mm
*Z* = 4	

### Data collection

**Table:**

Oxford Gemini E CCD diffractometer	*R*_int_ = 0.041
graphite	θ_max_ = 28.7°, θ_min_ = 3.7°
ω scans	*h* = −16→16
8306 measured reflections	*k* = −14→12
3531 independent reflections	*l* = −15→11
1723 reflections with *I* > 2σ(*I*)	

### Refinement

**Table:**

Refinement on *F*^2^	Primary atom site location: structure-invariant direct methods
Least-squares matrix: full	Secondary atom site location: difference Fourier map
*R*[*F*^2^ > 2σ(*F*^2^)] = 0.039	Hydrogen site location: inferred from neighbouring sites
*wR*(*F*^2^) = 0.079	H-atom parameters constrained
*S* = 0.78	*w* = 1/[σ^2^(*F*_o_^2^) + (0.0359*P*)^2^] where *P* = (*F*_o_^2^ + 2*F*_c_^2^)/3
3531 reflections	(Δ/σ)_max_ < 0.001
199 parameters	Δρ_max_ = 0.24 e Å^−^^3^
0 restraints	Δρ_min_ = −0.22 e Å^−^^3^

### Special details

**Table:**

Geometry. All e.s.d.'s (except the e.s.d. in the dihedral angle between two l.s. planes) are estimated using the full covariance matrix. The cell e.s.d.'s are taken into account individually in the estimation of e.s.d.'s in distances, angles and torsion angles; correlations between e.s.d.'s in cell parameters are only used when they are defined by crystal symmetry. An approximate (isotropic) treatment of cell e.s.d.'s is used for estimating e.s.d.'s involving l.s. planes.
Refinement. Refinement of *F*^2^ against ALL reflections. The weighted *R*-factor *wR* and goodness of fit *S* are based on *F*^2^, conventional *R*-factors *R* are based on *F*, with *F* set to zero for negative *F*^2^. The threshold expression of *F*^2^ > σ(*F*^2^) is used only for calculating *R*-factors(gt) *etc*. and is not relevant to the choice of reflections for refinement. *R*-factors based on *F*^2^ are statistically about twice as large as those based on *F*, and *R*-factors based on ALL data will be even larger.

### Fractional atomic coordinates and isotropic or equivalent isotropic displacement parameters (Å^2^)

**Table:**

	*x*	*y*	*z*	*U*_iso_*/*U*_eq_	
Cl1	0.85651 (5)	−0.90729 (5)	0.04716 (6)	0.0721 (2)	
P1	0.80969 (4)	−0.75005 (5)	−0.05171 (5)	0.04255 (16)	
C6	0.60540 (16)	−0.85615 (17)	−0.10376 (16)	0.0369 (5)	
C12	0.89415 (16)	−0.63352 (18)	0.14784 (19)	0.0462 (6)	
H12	0.8979	−0.7154	0.1783	0.055*	
C5	0.49261 (17)	−0.86465 (19)	−0.09499 (18)	0.0444 (6)	
C1	0.66389 (14)	−0.76001 (17)	−0.03965 (15)	0.0357 (5)	
C17	0.79619 (16)	−0.46293 (19)	−0.11219 (18)	0.0470 (6)	
H17	0.7693	−0.5296	−0.1572	0.056*	
C16	0.84215 (14)	−0.49050 (17)	−0.00446 (17)	0.0335 (5)	
C15	0.88282 (15)	−0.38650 (17)	0.06173 (19)	0.0403 (5)	
C14	0.92955 (17)	−0.4102 (2)	0.1698 (2)	0.0509 (6)	
H14	0.9573	−0.3424	0.2127	0.061*	
C7	0.65511 (18)	−0.94555 (18)	−0.17321 (17)	0.0468 (6)	
H7	0.7291	−0.941	−0.1808	0.056*	
C4	0.44061 (17)	−0.7756 (2)	−0.02860 (19)	0.0532 (6)	
H4	0.3663	−0.7801	−0.0242	0.064*	
C2	0.60878 (16)	−0.67670 (18)	0.02485 (17)	0.0454 (5)	
H2	0.6467	−0.6145	0.0665	0.054*	
C20	0.87568 (16)	−0.26148 (18)	0.0175 (2)	0.0511 (6)	
H20	0.9025	−0.1932	0.0605	0.061*	
C13	0.93470 (16)	−0.5302 (2)	0.21200 (18)	0.0526 (6)	
H13	0.9652	−0.5442	0.2839	0.063*	
C19	0.83054 (17)	−0.2391 (2)	−0.0863 (2)	0.0593 (6)	
H19	0.8263	−0.1559	−0.1139	0.071*	
C9	0.4862 (2)	−1.0482 (2)	−0.2178 (2)	0.0663 (7)	
H9	0.4475	−1.1132	−0.2548	0.08*	
C3	0.49639 (18)	−0.6832 (2)	0.02937 (19)	0.0551 (6)	
H3	0.4602	−0.6243	0.072	0.066*	
C11	0.84924 (15)	−0.61702 (16)	0.04156 (17)	0.0352 (5)	
C18	0.79022 (18)	−0.3405 (2)	−0.1522 (2)	0.0580 (6)	
H18	0.7592	−0.3247	−0.2235	0.07*	
C8	0.5971 (2)	−1.0381 (2)	−0.22923 (19)	0.0602 (7)	
H8	0.6314	−1.0951	−0.2754	0.072*	
C10	0.43458 (19)	−0.9638 (2)	−0.1532 (2)	0.0589 (7)	
H10	0.3606	−0.9709	−0.147	0.071*	

### Atomic displacement parameters (Å^2^)

**Table:**

	*U*^11^	*U*^22^	*U*^33^	*U*^12^	*U*^13^	*U*^23^
Cl1	0.0613 (4)	0.0341 (3)	0.1182 (6)	0.0056 (3)	−0.0207 (4)	0.0046 (3)
P1	0.0398 (3)	0.0334 (3)	0.0549 (4)	−0.0021 (3)	0.0067 (2)	−0.0070 (3)
C6	0.0423 (13)	0.0346 (10)	0.0333 (13)	−0.0041 (10)	−0.0034 (10)	0.0071 (10)
C12	0.0440 (13)	0.0444 (12)	0.0503 (16)	−0.0002 (11)	0.0027 (11)	0.0071 (11)
C5	0.0425 (13)	0.0484 (12)	0.0412 (14)	−0.0072 (11)	−0.0072 (11)	0.0142 (11)
C1	0.0362 (11)	0.0328 (10)	0.0380 (12)	−0.0014 (10)	0.0018 (9)	0.0063 (10)
C17	0.0461 (13)	0.0416 (11)	0.0529 (15)	−0.0009 (10)	0.0000 (11)	0.0017 (11)
C16	0.0280 (11)	0.0334 (10)	0.0393 (13)	−0.0003 (9)	0.0025 (9)	−0.0014 (9)
C15	0.0331 (12)	0.0357 (11)	0.0527 (16)	−0.0050 (9)	0.0097 (11)	−0.0059 (10)
C14	0.0430 (13)	0.0547 (13)	0.0550 (16)	−0.0107 (12)	0.0028 (11)	−0.0173 (12)
C7	0.0523 (14)	0.0397 (11)	0.0479 (15)	−0.0039 (11)	−0.0019 (11)	−0.0005 (11)
C4	0.0331 (12)	0.0712 (16)	0.0551 (15)	−0.0007 (12)	0.0019 (11)	0.0176 (13)
C2	0.0436 (13)	0.0438 (12)	0.0490 (15)	0.0001 (11)	0.0050 (11)	−0.0024 (11)
C20	0.0451 (12)	0.0332 (11)	0.0761 (18)	−0.0052 (11)	0.0132 (12)	−0.0090 (12)
C13	0.0489 (14)	0.0661 (15)	0.0416 (15)	−0.0051 (13)	−0.0094 (11)	−0.0038 (12)
C19	0.0557 (14)	0.0374 (12)	0.086 (2)	0.0037 (12)	0.0155 (14)	0.0115 (14)
C9	0.081 (2)	0.0539 (15)	0.0604 (19)	−0.0191 (15)	−0.0293 (15)	0.0065 (13)
C3	0.0475 (14)	0.0598 (14)	0.0590 (17)	0.0084 (12)	0.0114 (12)	−0.0032 (13)
C11	0.0297 (11)	0.0346 (11)	0.0414 (14)	−0.0005 (9)	0.0030 (10)	−0.0017 (10)
C18	0.0612 (16)	0.0551 (14)	0.0568 (17)	0.0069 (13)	−0.0050 (12)	0.0136 (13)
C8	0.0814 (19)	0.0456 (13)	0.0519 (16)	−0.0036 (14)	−0.0105 (14)	−0.0060 (12)
C10	0.0493 (15)	0.0670 (15)	0.0583 (18)	−0.0176 (14)	−0.0157 (13)	0.0184 (14)

### Geometric parameters (Å, °)

**Table:**

Cl1—P1	2.0867 (8)	C14—H14	0.93
P1—C1	1.8293 (18)	C7—C8	1.360 (3)
P1—C11	1.8309 (18)	C7—H7	0.93
C6—C5	1.415 (3)	C4—C3	1.356 (3)
C6—C7	1.414 (3)	C4—H4	0.93
C6—C1	1.436 (2)	C2—C3	1.403 (3)
C12—C11	1.368 (3)	C2—H2	0.93
C12—C13	1.401 (3)	C20—C19	1.351 (3)
C12—H12	0.93	C20—H20	0.93
C5—C4	1.403 (3)	C13—H13	0.93
C5—C10	1.422 (3)	C19—C18	1.396 (3)
C1—C2	1.370 (2)	C19—H19	0.93
C17—C18	1.366 (3)	C9—C10	1.356 (3)
C17—C16	1.407 (3)	C9—C8	1.397 (3)
C17—H17	0.93	C9—H9	0.93
C16—C15	1.420 (2)	C3—H3	0.93
C16—C11	1.433 (2)	C18—H18	0.93
C15—C14	1.406 (3)	C8—H8	0.93
C15—C20	1.410 (3)	C10—H10	0.93
C14—C13	1.352 (3)		
			
C1—P1—C11	103.26 (9)	C5—C4—H4	119.3
C1—P1—Cl1	99.06 (6)	C1—C2—C3	121.38 (19)
C11—P1—Cl1	101.38 (7)	C1—C2—H2	119.3
C5—C6—C7	117.98 (19)	C3—C2—H2	119.3
C5—C6—C1	118.65 (19)	C19—C20—C15	121.2 (2)
C7—C6—C1	123.35 (18)	C19—C20—H20	119.4
C11—C12—C13	121.68 (18)	C15—C20—H20	119.4
C11—C12—H12	119.2	C14—C13—C12	120.2 (2)
C13—C12—H12	119.2	C14—C13—H13	119.9
C4—C5—C6	119.33 (19)	C12—C13—H13	119.9
C4—C5—C10	121.5 (2)	C20—C19—C18	120.1 (2)
C6—C5—C10	119.2 (2)	C20—C19—H19	119.9
C2—C1—C6	119.30 (17)	C18—C19—H19	119.9
C2—C1—P1	122.45 (14)	C10—C9—C8	120.5 (2)
C6—C1—P1	118.21 (14)	C10—C9—H9	119.7
C18—C17—C16	121.5 (2)	C8—C9—H9	119.7
C18—C17—H17	119.3	C4—C3—C2	119.8 (2)
C16—C17—H17	119.3	C4—C3—H3	120.1
C17—C16—C15	117.73 (18)	C2—C3—H3	120.1
C17—C16—C11	123.52 (18)	C12—C11—C16	119.01 (17)
C15—C16—C11	118.75 (18)	C12—C11—P1	123.34 (14)
C14—C15—C20	121.36 (19)	C16—C11—P1	117.34 (15)
C14—C15—C16	119.43 (18)	C17—C18—C19	120.3 (2)
C20—C15—C16	119.2 (2)	C17—C18—H18	119.9
C13—C14—C15	120.92 (19)	C19—C18—H18	119.9
C13—C14—H14	119.5	C7—C8—C9	120.3 (2)
C15—C14—H14	119.5	C7—C8—H8	119.8
C8—C7—C6	121.4 (2)	C9—C8—H8	119.8
C8—C7—H7	119.3	C9—C10—C5	120.5 (2)
C6—C7—H7	119.3	C9—C10—H10	119.7
C3—C4—C5	121.44 (19)	C5—C10—H10	119.7
C3—C4—H4	119.3		
			
C7—C6—C5—C4	−178.67 (18)	P1—C1—C2—C3	177.53 (15)
C1—C6—C5—C4	3.0 (3)	C14—C15—C20—C19	−179.9 (2)
C7—C6—C5—C10	1.7 (3)	C16—C15—C20—C19	0.3 (3)
C1—C6—C5—C10	−176.61 (17)	C15—C14—C13—C12	0.6 (3)
C5—C6—C1—C2	−2.3 (3)	C11—C12—C13—C14	0.4 (3)
C7—C6—C1—C2	179.54 (18)	C15—C20—C19—C18	−0.2 (3)
C5—C6—C1—P1	179.99 (13)	C5—C4—C3—C2	−1.0 (3)
C7—C6—C1—P1	1.8 (2)	C1—C2—C3—C4	1.8 (3)
C11—P1—C1—C2	2.03 (18)	C13—C12—C11—C16	−0.9 (3)
Cl1—P1—C1—C2	106.09 (15)	C13—C12—C11—P1	172.55 (16)
C11—P1—C1—C6	179.72 (14)	C17—C16—C11—C12	−179.13 (19)
Cl1—P1—C1—C6	−76.22 (14)	C15—C16—C11—C12	0.5 (3)
C18—C17—C16—C15	−0.3 (3)	C17—C16—C11—P1	7.0 (3)
C18—C17—C16—C11	179.33 (19)	C15—C16—C11—P1	−173.42 (14)
C17—C16—C15—C14	−179.87 (18)	C1—P1—C11—C12	100.58 (18)
C11—C16—C15—C14	0.5 (3)	Cl1—P1—C11—C12	−1.68 (18)
C17—C16—C15—C20	0.0 (3)	C1—P1—C11—C16	−85.83 (16)
C11—C16—C15—C20	−179.64 (18)	Cl1—P1—C11—C16	171.91 (14)
C20—C15—C14—C13	179.1 (2)	C16—C17—C18—C19	0.3 (3)
C16—C15—C14—C13	−1.1 (3)	C20—C19—C18—C17	0.0 (3)
C5—C6—C7—C8	−0.7 (3)	C6—C7—C8—C9	−1.0 (3)
C1—C6—C7—C8	177.49 (18)	C10—C9—C8—C7	1.7 (3)
C6—C5—C4—C3	−1.5 (3)	C8—C9—C10—C5	−0.7 (3)
C10—C5—C4—C3	178.2 (2)	C4—C5—C10—C9	179.4 (2)
C6—C1—C2—C3	−0.1 (3)	C6—C5—C10—C9	−1.0 (3)

### Hydrogen-bond geometry (Å, °)

**Table:**

*D*—H···*A*	*D*—H	H···*A*	*D*···*A*	*D*—H···*A*
C12—H12···Cl1	0.93	2.58	3.128 (2)	118
